# Preclinical Remodeling of Human Prostate Cancer through the PTEN/AKT Pathway

**DOI:** 10.1155/2012/419348

**Published:** 2012-02-21

**Authors:** Marco A. De Velasco, Hirotsugu Uemura

**Affiliations:** ^1^Department of Urology, Kinki University School of Medicine, 377-2 Ohno-Higashi, Osaka-Sayama, Osaka 589-8511, Japan; ^2^Department of Genome Biology, Kinki University School of Medicine, Osaka-Sayama, Osaka 589-8511, Japan

## Abstract

Knowledge gained from the identification of genetic and epigenetic alterations that contribute to the progression of prostate cancer in humans is now being implemented in the development of functionally relevant translational models. GEM (genetically modified mouse) models are being developed to incorporate the same molecular defects associated with human prostate cancer. Haploinsufficiency is common in prostate cancer and homozygous loss of *PTEN* is strongly correlated with advanced disease. In this paper, we discuss the evolution of the *PTEN* knockout mouse and the cooperation between *PTEN* and other genetic alterations in tumor development and progression. Additionally, we will outline key points that make these models key players in the development of personalized medicine, as potential tools for target and biomarker development and validation as well as models for drug discovery.

## 1. Introduction

A major breakthrough in the developmental strategy for the treatment of prostate cancer came through in 1941 when Huggins discovered that metastatic prostate cancer responds to androgen ablation thus ushering in a new era in the treatment of prostate cancer therapy [1]. Androgen deprivation therapy remains the most powerful treatment for advance prostate cancer and newer generation androgen deprivation therapies (ADTs) that more effectively inhibit AR signaling are rapidly being developed and approved for patients with metastatic CRPC. However, therapeutic effects are short lived and eventually all patients succumb to the disease [2]. The prognosis for men with CRPC is bleak as currently available approved treatments only provide marginal benefit and systemic treatments for metastatic CRPC are primarily approved for the management of symptoms [3, 4]. Recently, cytotoxic treatments such as the combinations of prednisone plus docetaxel or cabazitaxel demonstrated modest improvements in extended survival but have yet to produce long-term benefits [5–7].

 A better understanding of the biology of prostate cancer has resulted in the identification of novel therapeutic targets and thus encouraged the development of new small molecule therapeutic agents that target quintessential factors that are now known to contribute to tumor growth, development and progression. A large number of novel therapeutics are currently undergoing clinical evaluation for the treatment of prostate cancer, and small molecule signal transduction inhibitors are a promising class of agents. These inhibitors have recently become standard therapy and have been FDA approved for the treatment of various solid cancers including renal, GIST, breast, pancreas, colon, and NSCLC and offer significant promise in prostate cancer [8–14].

 The development of an effective treatment strategy to treat advanced prostate cancer has been challenging due in fact to the heterogeneity of the disease. Complex genomic aberrations targeting multiple genes through mutation, changes in copy number, and methylation make patient selection difficult for the use of targeted molecular inhibitors [15]. The first steps have already been taken to identify some of the genetic alterations that lead to perturbed cell signaling pathways that contribute to tumor development and progression. However, developing effective therapeutic strategies will require relevant preclinical models of prostate cancer to identify and validate therapeutic targets and biomarkers.

## 2. Preclinical Modeling for Prostate Cancer

Traditional medical intervention treatments for prostate cancer are based on data from epidemiological, clinical, or evidence-based medicine. However, this model is optimized for a large population and not for any one particular individual. The recent trend in medicine is to employ a personalized treatment approach that is based on molecular profiling to determine the best treatment strategy for a particular individual. This approach requires the development of new high-throughput technologies to decipher and interrogate tumors at various molecular levels and integrates resources from various specialized fields into one system to discover, coordinate, and extract a strategic approach derived from multidimensional input data.

Cancer is a complex heterogeneous disease that involves genetic events that lead to the disruption of multiple signaling networks. Consequently, multiple cellular processes within the tumor microenvironment within a host system are involved and may be influenced by any number of environmental factors over a period of time. Basically, the “one gene, one protein, one function” hypothesis is outdated and not applicable for systems biology. Until recently, preclinical models for prostate cancer have been limited largely due to the lack of animal models that develop spontaneous prostate tumors in a manner similar to humans. Spontaneous animal models such as dog and rat have been used extensively to study hormonal carcinogenesis but are impractical and do not represent a feasible model for preclinical efficacy evaluation [16, 17]. Mouse xenograft models derived from human prostate tumor cell have been developed and used extensively in academia and pharmaceutical industry. Although the number of human prostate cancer cell lines is limited, the convenience and low cost have made the xenograft model popular for gene validation and compound testing [18, 19].

During a 10-year period (1991–2000) big pharma companies in the United States and Europe reported that only 5 out of 100 drugs passing preclinical testing achieved drug approval success [20]. The majority of attrition (70%) occurred in Phase II trials with lack-of-efficacy accounting for approximately 30% of failures. Lack of treatment efficacy in Phase II and III trials has been attributed to unpredictable preclinical models [20, 21]. Pharmaceutical companies routinely use human prostate cancer cell xenografts during the preclinical testing phase to evaluate new drug efficacy. However, only three human prostate cancer cells lines (PC3, DU145, and LNCaP) account for vast majority of cells used in prostate cancer drug efficacy screens. The use of these cells to study human prostate cancer *in vivo* is inadequate as they lack many key features found in autochthonous cancers. While xenograft models (ectopic or orthotopic) may have value in certain situations, they are inappropriate and bear little relevance to human prostate cancer.

With the development of GEM (genetically engineered mice), preclinical modeling for prostate cancer has evolved significantly in the last 15 years [16–23]. These models consist of mice that have been designed to deliberately inhibit or express a particular gene function through the introduction of foreign DNA. Today, GEM have become quite sophisticated and allow for any combination of tissue-specific expression of oncogenes as well as conditional, tissue-specific deletion of tumor suppressors. The value of GEM modeling relies on the development of transgenic mice that possess most of the clinicopathological and molecular characteristics of human prostate cancer.

## 3. Biology of Prostate Cancer and PTEN

Prostate cancer progression follows a series of defined states characterized by molecular changes associated with disease progression. The heterogeneity of prostate cancer has prevented clear identification and correlation of critical genetic events contributing to disease progression and treatment resistance. However, there are constant genetic alterations frequently present in prostate cancer such as the loss of *PTEN* function. *PTEN*, located on chromosome 10 (10q23), is a tumor suppressor gene that is broadly expressed during development and adulthood and is essential for embryogenesis [24]. *PTEN* encodes a dual lipid and protein phosphatase that functions as an inhibitor of PIP3 [25]. Accumulation of PIP3 then mimics the effect of phosphatidylinositol 3-kinase (PI3K) activation resulting in the activation of downstream effectors including Akt. Activation of Akt phosphorylates various physiological substrates that results in the stimulation of cell cycle progression, survival, migration, and metabolism [24–27].

*PTEN* function lost through mutations, deletions, or promoter methylation silencing occurs at a high frequency in many primary and metastatic human cancers in humans and is the second most commonly tumor suppressor gene after *p53* [24, 26–28]. Current estimates suggest that PI3K/Akt/mTOR signaling is upregulated in 30–50% of prostate cancers, often through loss of *PTEN *function [29, 30]. Clinical findings have demonstrated that biallelic deletion of *PTEN* correlates with disease-specific mortality and is associated with Akt and AR deregulation [30–32]. Published reports have shown that heterozygous loss of *PTEN* occurs in as many as 70–80% of primary tumors, and complete inactivation occurs in 20% of primary tumors and 63% of metastasis [29, 33, 34]. A more recent report showed that copy-number alterations (CNAs) in prostate cancer were present in 42% of primary tumors and 100% of metastases [35].

 It has been widely recognized that AR signaling remains important even in the presence of reduced androgen levels and thus remains a major target for targeted therapeutic interventions [36]. Clinically, the deletion of PTEN and AR expression has been significantly correlated to cancer-specific mortality in patients with CRPC [31]. Reports suggest that PI3K through AKT may play an important role in upregulating AR protein expression in the absence of PTEN [37, 38]. PTEN can modulate AR activity directly or through PI3K/Akt signaling pathways; however, levels of AR are often heterogeneous in late-stage disease [39–41]. Evidence from published reports has now shown that alterations in AR develop with sequential hormonal ablation therapies and tumor progression [42, 43]. Also, reciprocal feedback regulation between AR and PTEN in prostate cancer initiates a series of molecular events that contribute to growth survival and differentiation and may thus participate in ADT resistance. It was recently demonstrated that loss of AR expression, in the absence of PTEN, can lead to downregulation of *Fkbp5* and PHLPP-mediated Akt inhibition resulting in increased cellular proliferation [44, 45]. Therefore, it is essential to develop and test new compounds that target known compensatory and survival pathways in advanced prostate cancer and identify new targets for possible interventions.

## 4. Traditional PTEN Knockout Mouse Models of Prostate Cancer

The strong implication of PTEN in prostate cancer progression in humans has prompted the development of genetically GEM models based on *PTEN* inactivation (see Table 1). Traditional knockout models of PTEN developed in the late 1990's were generated by deleting exons 4, or 4 and 5 of the *PTEN* gene, which codes for the entire PTEN-phosphatase domain and part of the two *α*-helix motifs flanking the catalytic core [46, 47]. Homozygous inactivation of *PTEN* results in normal appearing, but nonviable embryos. Heterozygous *PTEN* knockout mice are born viable to develop prostatic intraepithelial neoplasia (PIN) in the prostate as well as a neoplasias in a number of organs including skin, colon, endometrium, liver, thyroid, and thymus [46, 47]. However, progression to malignant adenocarcinoma is not observed in heterozygous mutants indicating that inactivation of one allele of *PTEN* is enough to initiate tumorigenesis but not progression. It is important to note that the viability of the mice is compromised by lymphoid proliferation and development of tumors arising in other organs such as intestines, mammary, thyroid, endometrial, and adrenal glands. 

 Increased phosphorylation of Akt occurs as a result of *PTEN* inactivation; however, it was uncertain whether hyperactivation of Akt was enough to drive tumor development in the prostate. To address this question, one group looked at the effects of Akt overexpression in the mouse prostate using the MPAKT transgenic mouse [57]. Overexpression of Akt1 in MPAKT transgenic mice results in the development of PIN in the ventral prostate but not cancer. Thus activation of Akt signaling alone in the presence of PTEN is insufficient to induce prostate cancer although the deletion of Akt1 but not Akt2 (Akt1 is the predominant isoform found in mouse prostate) was sufficient to suppress the development of high-grade PIN lesions in *PTEN^+/-^* mice [54, 58]. These findings not only cement the role of PTEN in early prostate carcinogenesis but also demonstrate the multifunctional role of PTEN in regulating other biological processes related to malignant transformation. Prostate cancer in humans displays a range of clinical phenotypes that develops over time as a result of gene alterations involving multiple regulatory pathways [59, 60]. In order to achieve clinically relevant models of human prostate cancer in mice, several investigators have sought to generate bigenic knockout mice that combine *PTEN* haploinsufficiency with other genetic alterations to further characterize the role of PTEN in prostate tumorigenesis.

 Alterations of *p53* and *retinoblastoma* (*Rb*) oncogenes correspond to prostate cancer progression in humans [61, 62]. One particular study used the TRAMP mouse model to investigate the cooperation between *PTEN* haploinsufficiency and abrogated function of the tumor suppressor genes *p53* and *Rb* in prostate cancer development [49]. The TRAMP mouse model is a first generation transgenic knockout and was one of the first mouse models to effectively induce the development of aggressive prostate tumors through the expression of large/small SV40 tumor antigens (T/tag) under the control of the prostate-specific rat probasin promoter [22]. The transforming activity of T/tag inactivates both p53 and Rb tumor suppressor proteins [63]. Prostate cancer progression in *PTEN^+/−^/TRAMP* mutant mice shows increased rates of tumor development and decreased survival compared to *PTEN^+/+^/TRAMP *mice. A different study used the *Ink4a/Arf^−/−^PTEN^+/−^* model to investigate the cooperation between PTEN haploinsufficiency and RB and p53 [50]. The *Ink4a/Arf *gene focu*s* regulates the tumor suppressor proteins RB and p53 through *p16^Ink4a^* and *p19^Arf^*, respectively [64]. *Ink4a/Arf^−/−^PTEN^+/−^* mice experienced a much faster rate of PIN development compared to *Ink4a/Arf^+/+^PTEN^+/−^* controls; however, these mice did not develop adenocarcinomas [50].

 Deletions of chromosome 12p11-13 (corresponding to *CDKN1B(p27/Kip1I0)*) have been identified in advanced human prostate cancer suggesting a tumor suppressor role for *p27(Kip1) *[48]. Loss of *p27(Kip1)* function has been implicated with prostate tumor recurrence and poor disease-free survival in humans [65, 66]. *p27^−/−^* mice develop enlarged hyperplastic prostates and increased fibromuscular stromal cells closely resembling benign prostatic hyperplasia (BPH) but fail to develop prostate cancer [67]. However, when these mice are bred with heterozygous *PTEN* mutant mice, all resulting *p27^−/−^/Pten^+/-^* mutant mice became susceptible to the development of invasive prostate adenocarcinomas [68]. These animal models have provided genetic evidence to show that collaboration between *PTEN* haploinsufficiency and inactivation of other tumor suppressor genes by either gain or loss of function promotes prostate cancer progression.

 Nkx3.1 is a transcription factor whose expression is androgen dependent and limited to the luminal cell compartments in prostate glandular tissue [69]. Although *Nkx3.1* mutations are not reported in humans, loss of Nkx3.1 protein expression is strongly correlated to CRPC and advanced stage prostate cancer [70, 71]. The cooperative function of *PTEN* and* Nkx3.1* haploinsufficiency was explored in a double knockout transgenic mouse model [51–53]. In this model, double heterozygous mutants demonstrate a propensity to develop invasive prostate adenocarcinoma after 12 months of age and frequently display iliac lymph node metastases. In contrast, *Nkx3.1* knockout mice only develop PIN lesions [72, 73]. Another interesting observation with *Nkx3.1^+/-^/PTEN^+/-^* mice is the ability for these mice to develop CRPC after castration.

 The *ERG* gene is frequently translocated to the *TMPRSS2* promoter region; the resulting TMPRSS2-ERG fusion protein is positively expressed in half of human prostate cancer cases [74–76]. Mice expressing the truncated ERG product from *TMPRSS2-ERGa*, under the control of the androgen-responsive region (*ARR2Pb*) probasin promoter (functionally analogous to the *TMPRSS2-ERGa* fusion product), only develop PIN [75]. In the presence of *PTEN *haploinsufficiency, overexpression of *ARR2Pb-ERG* results in the progression of PIN lesions to prostatic adenocarcinoma [55]. This model has confirmed that two common critical events, concomitant loss of *PTEN* and *EGR* genetic rearrangement, accelerate initiation and progression in human prostate adenocarcinoma. Stat3 has been implicated in the promotion and progression of human prostate cancer [77]. Transgenic mice designed to constitutively express Stat3 under the control of *ARR2Pb* develop PIN but fail to progress to malignant adenocarcinoma; however, when crossed with *PTEN^+/−^* mutant mice, the resultant double knockouts develop invasive adenocarcinomas [56]. Phosphorylated Stat3 expression was potentiated by the loss of PTEN and subsequent overexpression of Akt. Collectively, these studies have shown the crucial relevance of “two hits” for the development of prostate adenocarcinoma and demonstrated how genetic alterations that play subtle roles in tumor initiation cooperate with *PTEN* haploinsufficiency to produce malignant phenotypes in mice similar to human prostate adenocarcinoma.

## 5. Conditional PTEN Knockout Mouse Models of Prostate Cancer

Development of conditional gene targeting by the Cre-LoxP system has significantly changed the landscape for transgenic mouse modeling research. In conditional mouse models, the target gene is flanked by LoxP cassettes and remains in the germline. Inactivation of this gene is controlled by Cre recombinase which catalyzes recombination between the two LoxP sites [78]. Orientation of the LoxP cassettes determines type of recombination to produce deletion, inversions, or chromosomal translocations [79]. Expression of Cre is dependent on transgene expression of a widespread or tissue-specific promoter. A variation of this system uses an inducible transgene promoter that is inactive until it is induced by an activating agent [80]. Conditional knockout models have the ability to induce the genetic mutation in the target tissue without affecting nontargeted cells. In this manner, both genes can be knocked out in the target cells while the rest of the mouse cells retain normal gene expression and function.

 Promoter selection is critical for targeting the prostate gland, and several have been characterized and well described in the literature [16, 81–83]. The most common promoters used in prostate-specific conditional targeting are the *prostate specific antigen-Cre* (*PSA^-Cre^*), *probasin-Cre* (*PB^-Cre^*), and *ARR2PB-Cre* (*PB^-Cre4^*) promoters [84–88]. The *mouse mammary tumor virus* (*MMTV^-Cre^*) promoter has also been used to conditional drive mutations in the prostate; however, its activity was not specific to the prostate gland [89]. Inducible promoters used for conditional targeting of the mouse prostate include *PSA^CreERT2^* and *Nkx3.1^CreERT2^*, both inducible with tamoxifen [90, 91]. Floxed PTEN mice have been developed by flanking exons 4, or 4 and 5 with LoxP cassettes [84, 92–94]. As in traditional knockouts, these sites correspond to the coding regions for the entire PTEN-phosphatase domain and portion of the two *α*-helix motifs flanking the catalytic core [46, 47].

 We and others have generated prostate-specific conditional mouse models of prostate cancer to better characterize full loss of *PTEN* gene expression and its effect on prostate tumor carcinogenesis, summarized in Table 2. Heterozygous *PTEN^loxp/+^* mice develop PIN in a manner similar to traditional heterozygous *PTEN* knockouts [84–86, 89]. However, *PTEN* inactivation under the control of *PSA^Cre^* or *PB^Cre4^* promoter in *PTEN^loxp/+^* mice is largely restricted to the prostate, and trace levels of *PTEN* deletion are seen in the seminal vesicles [84–86]. Complete inactivation of *PTEN* in traditional knockouts results in embryonic lethality thus limiting the characterization of total *PTEN* inactivation. Development of PIN occurs quickly in homozygous PTEN knockout mice ranging from 6 to 16 weeks of age, and latency to the development of prostate adenocarcinoma varies from 9 to 24 weeks [84–86, 89]. Locally invasive disease is present in these models and some mice develop metastases to iliac lymph nodes, and occasionally lung [84–86]. A clinically relevant feature of prostate-specific *PTEN* conditional knockout mice is the sensitivity to androgen ablation and the ability to develop CRPC [85, 91].

 Altogether, these studies have shown that prostate-specific conditional *PTEN* knockout mice share many features seen in human prostate cancer. Biallelic inactivation of *PTEN* leads to hyperproliferation that is followed by the development of PIN which eventually progresses to locally invasive adenocarcinoma and eventual metastases. Moreover, tumors are initially responsive to androgen ablation and develop into CRPC. Besides histopathological similarities, tumors from these mice also share molecular profiles similar to human prostate cancer [96]. Inducible variations of the prostate-specific conditional knockout model provide spatiotemporal control of induced mutagenesis [90, 91]. The ability to incorporate bigenic gene alterations to mice with conditional *PTEN* haploinsufficiency makes it a relevant preclinical model to study the epigenetic events or LOH that lead to disease progression.

## 6. PTEN Knockout Mice as Drug Targeting Models

GEM models offer several unique advantages over the xenograft model. The first and probably most important feature is that through controlled gene disruption, these mice can be manipulated to develop prostate cancer from phenotypically normal cells, thus encompassing the whole spectrum of tumor carcinogenesis. Secondly, tumors develop *in situ* taking into account all the components involved in the carcinogenesis process, including interactions with all tumor microenvironment factors that can promote tumor development. Another key feature of these mice is that they retain an intact immune system, thus incorporating all the important components of innate and acquired immunity. Lastly, as in humans, tumors in these mice show heterogeneity, a key feature of cancer.

 Despite all of the advantages over xenograft models, concerns exist whether tumors arising from GEM are homologous to human prostate cancers. Compared to the human prostate which is divided into zones, the mouse prostate develops as a lobular structure consisting of the anterior, dorsal, lateral, and ventral lobes [97]. Some believe that the dorsolateral lobes of the mouse prostate are the most similar to the human peripheral zone, which is the region where most cancers arise [96–98]. However, the Bar Harbor pathology panel for genetically modified mouse models of prostate cancer had the consensus opinion that there is no direct relationship between the lobes of mouse prostates and human prostate zones [97]. Nevertheless, GEM offer a unique tool for biomedical research in the understanding of biochemical and disease pathways and the development of new therapeutic strategies through new target and biomarker discovery and validation.

 The evolution of newer generation transgenic mice based on the conditional mutation, deletion, or insertion of single or multiple targeted genes is becoming an attractive model for researchers in academia and industry. As a result, mice develop tumors which feature many similarities to human prostate cancer including various pathological and molecular characteristics [84–86, 96]. Since tumors in these mice arise from normal tissues, preclinical trials can be designed to target specific points during tumor development that take advantage of the windows of opportunity provided. A developing paradigm for new treatments strategies involves the use of combination-targeted therapies. Tumor growth is not dependent on one particular signaling pathway, rather, it is an orchestrated event that is driven by complex feedback loops from crosstalk between multiple signaling pathways. PTEN and bigenic knockout mice are excellent models to investigate the preclinical therapeutic effects from combinatorial treatment strategies. Treatment strategies can be designed as either horizontal or vertical to inhibit targets involved in altered signaling pathways resulting from PTEN inactivation.

 PI3K/Akt/mTOR inhibitors are currently being evaluated in various tumor types. mTORC1 inhibitors such as rapamycin and rapalogs have demonstrated limited success as single agent treatments [28, 99–101]. This lack of efficacy is attributed to the inability to maintain reduced levels of phosphorylated 4E-BPs resulting from upregulation of Akt through the loss of the S6K to IRS-1 negative feedback regulation loop [100, 102, 103]. However, published reports also suggest that crosstalk between RAS/RAF/MEK signaling after mTOR inhibition results in resistance to mTOR inhibitors. Humans with advanced prostate cancer treated with RAD001 show schedule-dependent increases of MAPK signaling activation [104]. Data from two independent studies conducted with *PTEN* knockout mice demonstrate that dual inhibition of PI3K/Akt/mTOR and MAPK signaling results in synergetic antitumor responses and is at least feasible in a preclinical setting [104, 105].

 Although Ras mutations in prostate cancer are infrequent, wild-type Ras is chronically activated in prostate cancer as a result of autocrine and paracrine growth factor stimulation [66, 106]. Upregulation of MAPK signaling in prostate cancer is likely due to overexpression of growth factor receptors. Several growth factor receptors including the epidermal growth factor receptor (EGFR) and insulin-like growth factor-1 receptor (IGF-1R) have been shown to be overexpressed in prostate cancer. EGFR belongs to the ErbB family of receptor tyrosine kinase proteins and is highly expressed in primary tumors and metastases. In prostate cancer, EGFR overexpression is associated with poor prognosis and the transition to CRPC status [107, 108]. It has also been shown that Ras activation can play a causal role in moving PCa cells towards decreased hormone dependence and an increased malignant phenotype [109]. The role of MAPK signaling, as a target for prostate cancer therapy, becomes complicated as others report that MAPK signaling may be inhibited in advanced prostate cancer due to the deletion of the *PTEN* [110–112]. Akt activation, through the deletion of *PTEN*, can result in the phosphorylation and inactivation of Raf-1 thus decreasing downstream signaling of MEK and ERK which then leads to the loss of cellular differentiation [111, 113]. Evidence of crosstalk between PI3K/Akt/mTOR and MAPK signaling pathways suggests that compensatory survival signaling exists in this network and could therefore be exploited therapeutically [114].

The transcription factor, signal transducer and activator of transcription 3 (Stat3), has been implicated in the growth and progression of several cancer types including prostate [77, 115–118]. Stat3 has been shown to directly and indirectly regulate the expression of genes required for proliferation and apoptosis and is also known to negatively regulate the expression of p53, stimulate tumor angiogenesis, and suppress antitumor immune responses [77, 119–121]. Stat3 has been shown to induce the metastatic behavior of prostate cancer cells *in vitro* and *in vivo *[77]. Activation of Stat3 occurs by the binding of various cytokines which become constitutively activated by their respective ligands by an autocrine and paracrine manner [120, 122]. Stat3 is also activated by growth factors as a downstream target of PI3K/Akt/mTOR and MAPK signaling through the phosphorylation of Ser-727 [121, 123]. Activated IL-6 has been shown to be elevated in the sera from patients with metastatic prostate cancer. In addition to activating Stat3, IL-6 can also induce MAPK activation through various distinct mechanisms [124–127]. Combined targeted inhibition of PI3K/Akt/mTOR, RAS/RAF/MEK, and JAK/STAT signaling may be a promising strategy for the treatment of prostate cancer and *PTEN* knockout mice should play an important role in the preclinical development and discovery of candidate agents.

## 7. PTEN Knockout Mice in Biomarker Discovery

To effectively treat human prostate cancer, one must be able to identify specific targets that drive molecular and cellular events to tumorigenesis. Cancer-related cellular processes are being studied to identify possible targets for new drug development and biomarker discovery. However, drug target and biomarker discovery using human samples is difficult and hampered by the amount of genetic variation among individuals as well as external influences (lifestyle and environmental factors) that contribute to the pathogenesis of the prostate cancer [128–130]. Furthermore, this requires the acquisition of large numbers of samples which is time consuming and may be difficult in many instances. Interspecies conservation of genomic aberrations across conserved regions of tumorigenesis provides an alternative approach to identify genes responsible for tumor developments and progression [15, 131]. Transgenic mice have lower biological variances and can be studied under controlled situations that better enable the detection of target molecules. Because of this, transgenic mouse models of prostate cancer, in particular *PTEN*-mutant mice, provide a unique opportunity for the discovery of novel targets.

## 8. Concluding Remarks

**Figure 1:**
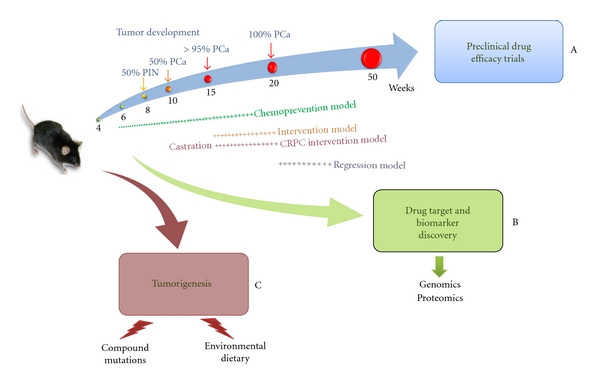
Modeling prostate cancer in the PTEN conditional knockout mouse model. (A) *PSA^Cre/^PTEN^loxP/loxP^* can be used to screen for tumor response against targeted therapies in chemoprevention, intervention, or regression models using noncastrated or castrated mice. (B) Comprehensive genomic and proteomic analyses can be performed in *PSA^Cre/^PTEN^loxP/+^*, *PSA^Cre/^PTEN^loxP/loxP^*, or bigenic mutants to identify candidate genes or proteins signatures aberrantly expressed between different pathologic, genomic, or temporal disease conditions. (C) Cooperation between genetic and nongenetic factors can be assessed in tumor development in both homozygous and heterozygous *PTEN*-conditional mutant mice.

Further advances in the treatment strategies for prostate cancer are dependent on the development, use, and incorporation of clinically relevant faithful animal models of human prostate cancer (Figure 1). Recent work on PTEN mouse models has helped characterize human prostate carcinogenesis. Although these models share amazing similarity to the pathobiology of human prostate cancer, differences between human and mouse kinetics, physiology, and metabolism must be considered. Despite these limitations, *PTEN* knockout mice will continue to be used to further characterize prostate carcinogenesis. The use of these models in preclinical drug, target, and biomarker discovery and development will increase and will most likely become a standard in drug discovery pipeline.

## Figures and Tables

**Table 1:** Traditional Pten knockout mouse models of prostate cancer.

Description	Gene knockout level	*Pten* mutation locus	Mouse strain	Phenotype	Castration resistance	Comments	Year	Ref.
*Pten^+/−^*	Single	Exons 4 and 5	129SvJy/C57BL/6	PIN	Not reported	*Pten^−/−^* progeny were nonviable; multiple organ neoplasia in *Pten^+/− ^* mutants	1998	[46]
*Pten^+/−^*	Single	Exon 5	129SvJy/C57BL/6	PIN	Not reported	*Pten^−/−^* progeny were nonviable; multiple organ neoplasia in *Pten^+/−^* mutants	1999	[47]
*Pten^+/−^/Cdkn1b^−/−^*	Compound	Exon 5	C57BL/6	Invasive adenocarcinoma	Not reported	Rapid progression of invasive carcinoma and decreased survival	2001	[48]
*PTEN^+/−^/TRAMP*	Compound	Exon 5	129SvJy/C57BL/6	Metastatic neuroendocrine carcinoma	Not reported	Increased rate of tumor development and metastases	2001	[49]
*Ink4a/Arf^−/−^/Pten^+/-^*	Compound	Exon 5	FVB/n/C57BL/6	PIN	Not reported	Early onset of PIN lesionsMultiple organ neoplasia and reduced tumor-free survival	2002	[50]
*Pten^+/-^/Nkx3.1^+/-^*	Compound	Exon 5	129SvJy/C57BL/6	Metastatic adenocarcinoma to lymph nodes	Yes	Mice developed adenocarcinomas in the dorsolateral prostate at 12 months and androgen independent phenotypes following castration	2003	[51–53]
*Pten^+/−^/Akt1^−/−^*	Compound	Exons 4 and 5	129SvJy/C57B6	PIN	Not reported	Akt deficiency attenuated PIN development	2006	[54]
*Pten^+/−^ PB-ERG*	Compound	Exons 4 and 5	129SvJy/C57BL/6	Invasive adenocarcinoma	Not reported	Overexpression of ERG cooperates and Pten haploinsufficiency leads to invasive adenocarcinoma and reduced cancer latency	2009	[55]
*ARR2Pb.Stat3C/ PTEN^+/−^*	Compound	Exon 5	FVB/n/C57BL/6	Invasive adenocarcinoma	Not reported	Increased incidence of AdCa in the ventral lobe	2011	[56]

**Table 2:** Conditional Pten knockout mouse models of prostate cancer.

Description	PTEN genetic manipulation	Gene knockout level	*Pten* mutation locus	Mouse strain	Phenotype	Castration resistance	Comments	Year	Ref.
*PB^Cre4^/Pten^loxp/loxp^*	Conditional	Single	Exons 4 and 5	129SvJy/C57BL/6	Homozygous deletion results in invasive adenocarcinoma and metastatic spread to lymph nodes	Not reported	*ARR2PB-Cre *promoter-driven; 100% of mice developed invasive carcinoma at 6 months	2003	[84]
*PB^Cre4^/Pten^loxp/loxp^*	Conditional	Single	Exon 5	C57BL/6/DBA2 /129/BALB/c	Homozygous deletion results in invasive adenocarcinoma and metastatic spread to lymph nodes	Yes	*ARR2PB-Cre *promoter-driven; Pin lesions develop at 6 weeks and invasive adenocarcimoa by 9 weeks	2003	[85]
*MMTVCre/PTEN^loxp/loxp^*	Conditional	Single	Exons 4 and 5	C57BL/6	Homozygous deletion results in invasive adenocarcinoma	Not reported	*MMTV-Cre *promoter-driven; focally invasive carcinoma at 10 weeks Mice die from lymphomas at 14 wks	2004	[89]
*PSA^Cre^/Pten^loxp/loxp^*	Conditional	Single	Exon 5	FVB/n/129Ola	Invasive adenocarcinoma	Not reported	*PSA-Cre* promoter-driven; all mice develop adenocarcinoma at 10–14 months with rare metastases	2005	[86]
*PSA^Cre^/Pten^loxp/loxp^*	Conditional	Single	Exons 4 and 5	C57BL/6	Homozygous deletion results in invasive adenocarcinoma and metastatic spread to lymph nodes	Yes	*PSA-Cre *promoter-driven; 50% incicence of adenocarcinoma at 10 weeks, lymph node metastasis >12 months	2012	[a]
*Pb-Cre4/Pten^loxP/loxP/^* *Trp53^loxP/loxP^*	Conditional	Compound	Exons 4 and 5	129SvJy/C57BL/6	Homozygous deletion results in invasive adenocarcinoma	Not reported	*ARR2PB-Cre *promoter-driven; invasive adenocarcinoma at 4–6 months with mean survival of 5 months	2005	[88]
*PB^Cre4^/Pten^+/loxP^/ FGF8b*	Conditional	Compound	Exon 5	C57BL/6/DBA2x 129/BALB/c	Homozygous deletion results in invasive adenocarcinoma and metastatic spread to lymph nodes	Not reported	*ARR2PB-Cre *promoter-driven; activation of *FGF8b and* heterozygous loss of *Pten *cooperate in the late-onset induction of metastatic prostate cancer with high incidence	2006	[87]
*PSA^CreERT2^/Pten^loxp/loxp^*	Inducible conditional	Single	Exons 4 and 5	129SvJy/C57BL/6/ FVB/n	Homozygous deletion results in invasive adenocarcinoma after tamoxifen treatment	Not reported	Tam-inducible *Cre-ERT2* recombinase under the control of the human* PSA *proximal promoter	2008	[90]
*Nkx3.1^CreERT2^/ Pten^loxp/loxp^*	Inducible conditional	Single	Exon 5	C57BL/6/129/Sv	Homozygous deletion results in invasive adenocarcinoma after tamoxifen treatment	Yes	Tam-inducible *Cre-ERT2* recombinase under the control of the *Nkx3.1 *promoter	2009	[91]

^a^De Velasco et al. [95].
